# Considering the missing science of retraining and maintenance in medical artificial intelligence, using ophthalmology as an exemplar

**DOI:** 10.1038/s41746-026-02463-2

**Published:** 2026-02-25

**Authors:** Ariel Yuhan Ong, Robbert R. Struyven, Alastair K. Denniston, David A. Merle, Justin Engelmann, Hyunmin Kim, Yukun Zhou, Pearse A. Keane, Ines Lains

**Affiliations:** 1 Institute of Ophthalmology, University College London, London, UK; 2 Moorfields Eye Hospital NHS Foundation Trust, London, UK; 3 NIHR Moorfields Biomedical Research Centre, London, UK; 4 Department of Ophthalmology, Massachusetts Eye and Ear, Harvard Medical School, Boston, MA USA; 5 NIHR Birmingham Biomedical Research Centre, Birmingham, UK; 6 Department of Applied Health Sciences, University of Birmingham, Birmingham, UK; 7 Birmingham Health Partners Centre for Regulatory Science and Innovation, Birmingham, UK

**Keywords:** Computational biology and bioinformatics, Mathematics and computing, Scientific community, Social sciences

## Abstract

Considerations around model retraining are standard practice in industry and non-healthcare sectors; however, this is much less well explored in medical artificial intelligence (AI). The problem is not only that models often fail to generalise, but that academia in particular does not have a systematic science of retraining to address this gap. This matters for building trustworthy models capable of making a lasting impact, rather than compounding as research waste. In this Perspective, we highlight three common challenges that constrain model retraining in medicine, and argue that academia must evolve beyond a focus on developing proofs-of-concept and world-first innovations to also recognise model retraining as scholarship. Drawing from case examples in ophthalmology, we call on stakeholders to consider not just how we build AI models, but how we should retrain, maintain, and share them.

## Introduction

Artificial intelligence (AI) has generated significant enthusiasm in medicine for its potential to transform clinical decision-making and patient care^1^, as well as accelerate scientific discovery^2^. However, despite the exponential growth of papers on AI models in biomedical research, implementation remains relatively limited. This gap, termed the ‘AI chasm’, is multifactorial in nature, comprising diverse aspects such as lack of transparency and trust, generalisability issues, regulatory science, and human factors^3^. While research into these areas is ongoing, other considerations about real-world use, such as plans for future-proofing these AI models, lag behind.

To date, most academic publications in healthcare have focused on developing or validating exciting new proof-of-concept models and world-first innovations, typically under tightly controlled conditions. This makes most AI models fragile across time, settings, populations, and imaging devices, given that what works well in a sterile laboratory setting often fares less well in the real world^4^. However, far fewer have tackled the practical challenges of retraining models across contexts, settings, and time, or the system-level implications (infrastructure, capacity, workflow) of doing so. The consequences differ in different contexts and settings, but addressing this is important for academic tools to have further value beyond their original publications and to become trustworthy models capable of making a lasting impact on scientific discovery rather than compounding as research waste.

In contrast, in non-healthcare sectors such as finance or retail, practices such as updating a model to maintain performance under changing conditions (e.g. a new but related use case or population) and versioning (i.e. maintaining and tracking multiple iterations of the model) have become well-established components of machine learning lifecycle management frameworks such as ‘machine learning operations (MLOps)’^5,6^. Retraining and fine-tuning are specific strategies within this broader paradigm, both involving updates to an existing model’s parameters using new data, but differing primarily in scope and extent rather than representing categorically distinct processes. These approaches are essential because shifts in data distributions (e.g. changes in consumer behaviour or market conditions) and software environments can quietly degrade model performance if not managed through structured lifecycle processes^5^. However, these developments typically occur in proprietary silos in commercial settings. MLOps remains relatively underdeveloped and less well-defined in academic healthcare research, where no standardised MLOps framework encompassing the regulatory, organisational, and infrastructural complexities exists thus far^7^. The lack of open discussion and information-sharing risks compounding cumulative knowledge loss, highlighting the need to tackle these challenges in academia so that the rest of the community can learn from this.

## The generalisability and retraining challenge

**Fig. 1:**
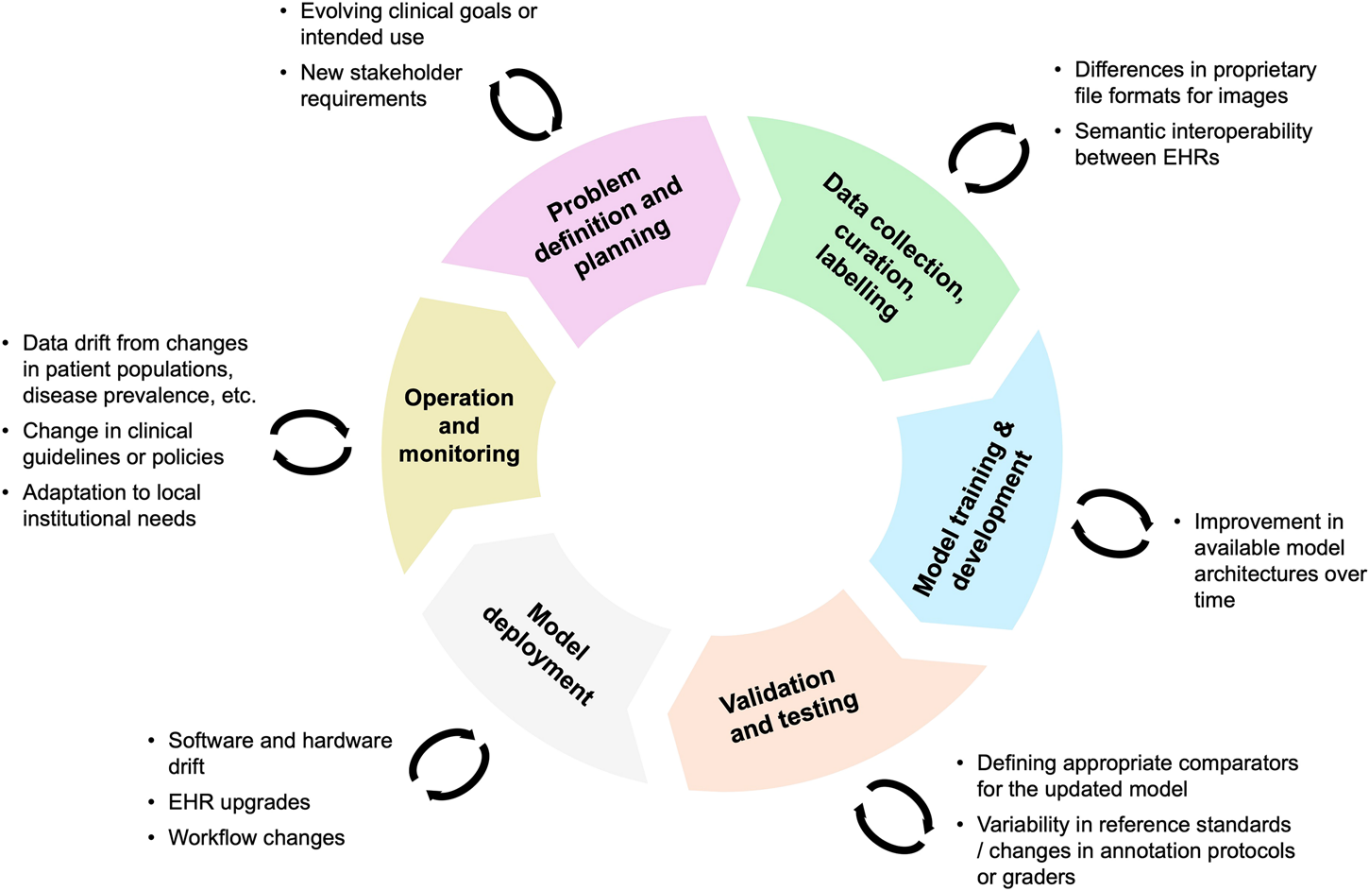
Challenges for AI model retraining across the AI lifecycle, with examples of common considerations that affect each phase. This ranges from interoperability issues from different data formats in the data collection phase, to improvements in model architectures over time for model development, to software and hardware drift in the model deployment phase and so on. Builds on the framework described by Tazbaz and Nicol (Digital Health Centre of Excellence, FDA)^41^.

In this Perspective, we outline the current challenges around model generalisability and retraining, and make the case for tackling these issues in academia. These challenges span the whole AI lifecycle (Fig. 1) and are particularly salient to fields that benefit from medical image analysis, such as radiology, pathology, and ophthalmology.

### Proprietary data formats and fragmented standards

These issues begin at the earliest stages of development with infrastructural challenges such as data export and preprocessing. Clinical imaging data is frequently stored in proprietary file formats (e.g. .fda (Topcon), .e2e (Heidelberg), .img (Zeiss) for ophthalmic images), and access is gated through Picture Archiving and Communication Systems (PACS) specific to each imaging device manufacturer. This means that researchers must often reverse-engineer proprietary or poorly documented data pipelines in order to extract basic image metadata. Some institutions even require multiple PACS, each focusing on a single manufacturer’s data, resulting in the fragmentation of patient data across multiple systems and challenging the FAIR principles of ‘findability, accessibility, interoperability, and reusability’^8^.

**Fig. 2:**
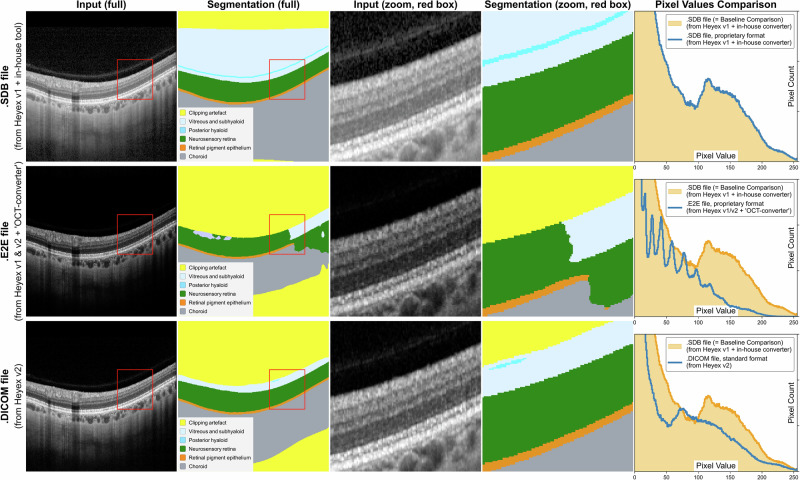
Example of how the imaging format affects the performance of algorithms. The same image was exported as a .sdb file (row 1), .e2e file exported with an OCT converter (row 2), and a DICOM file (row 3). As shown in the last column, these demonstrate subtle pixel-level differences, which can result in errors when a segmentation model trained on files exported in a specific manner is used on other file types. In the second row, pixel differences result in the segmentation of the retina (green region) being discontinuous. In the third row, the posterior hyaloid (blue line) is poorly visible in the input scan and was therefore not appropriately segmented. In contrast, the first row is correctly segmented.

Despite the clear need for harmonised imaging standards, progress has been slow due to the lack of direct incentives for change from imaging hardware and software manufacturers. Data standards such as the Digital Imaging and Communications in Medicine (DICOM) are more widespread among radiology imaging manufacturers, but vendor-specific implementations can introduce proprietary tags and inconsistent metadata usage that influence interoperability^9^. Uptake of DICOM standards by ophthalmic imaging manufacturers remains more variable, as does the degree of conformance^10^. Even after the adoption of DICOM standards, different software versions, inbuilt image processing settings, and methods of exporting images can continue to influence image appearance (Fig. 2). In pathology, whole-slide imaging systems use incompatible file types (e.g. .svs, .ndpi, .mrxs) and annotation formats that impede data sharing^11^. In cardiology, echocardiography images are often stored in proprietary formats tied to individual ultrasound vendors, with measurements embedded in inaccessible metadata or custom XML schemas^12^.

Beyond data, the computational environment also risks incurring technical debt. Software drift (e.g. updated programming libraries, software dependencies, or the broader system environment) and hardware drift (e.g. GPU upgrades) can influence reproducibility. Upgrades to imaging devices or image management platforms may introduce a further source of drift. For example, the transition from Heyex 1 to Heyex 2 (Heidelberg Engineering GmbH, Heidelberg, Germany)^13^ in ophthalmology, while ostensibly providing new features and improved security, influenced the underlying database schema, and produced DICOMs that vary in metadata, image resolution, and segmentation outputs (Fig. 2). In addition, migrating large volumes of historical patient data from one system to the next presents additional complexities and time demands for researchers aiming to ensure continuity in model performance and reproducibility.

All these issues undermine cross-institutional AI development, deployment, and retraining.

### Data drift

Data drift occurs when an AI model underperforms due to a mismatch between the data on which it was developed and the data on which it is deployed^14,15^. Causes of data drift include (i) temporal drift due to changes over time, and (ii) setting drift, which occurs when a model is deployed in a different setting than the data on which it is trained. Both of these can be attributed to changes in populations (e.g. shifts in patient demographics, disease prevalence, comorbidities), workflow patterns, and testing protocols^14^, or because models trained and tuned to perfection in laboratory settings on clean datasets fare less well outside controlled environments, particularly as a spectrum of diseases, disease phenotypes, and image quality is more typical of real-world clinical practice.

Beyond imaging, models developed using other modalities such as electronic health records (EHR) and genomics present analogous challenges, reflecting instability in medical data generation processes rather than modality-specific limitations. In addition to the common sources of data drift described above, for EHR-based models (which typically comprise a combination of free-text and structured data), variation in coding conventions and documentation or medical practices can also drive distributional shift^16,17^. In genomics, changes in sequencing platforms and reference annotations can similarly affect interoperability between datasets^18^, which can complicate model reuse and generalisation.

Ironically, in addition to necessitating retraining, data drift also acts as a barrier to this process. Data drift turns retraining from a simple update into an action that requires a complex lifecycle because of the diagnostic challenge it presents (identifying the nature of the drift so as to address it), the resource burden (collecting and labelling representative data for retraining), and the need for careful validation (including avoiding the risk of ‘catastrophic forgetting’)^19,20^ to ensure that the updated model continues to perform safely and effectively.

The chronic under-reporting of population stratification in publicly available datasets, which are often used as training and test sets for medical AI, compounds this challenge^21^. A recent scoping review of regulator-approved AI tools for ophthalmic image analysis found that demographics were poorly reported across clinical evaluation studies (age recorded in 52%, sex 51%, ethnicity 21%), many of which were tested on publicly available datasets, with minimal reporting of subgroup performance obscuring subgroup-specific failure modes^22^. Training or testing on highly curated datasets further exacerbates this issue through spectrum bias^23^, wherein model performance is artificially inflated through the exclusion of poor quality images, borderline/ambiguous images, or exclusive selection of the pathology of interest rather than that reflecting the real-world spectrum of disease^24^. This can also lead to demographic bias^25^. This fundamental lack of insight into the data’s provenance and composition means there is a significant and unquantifiable risk that these models will underperform or exhibit harmful biases when deployed into heterogeneous real-world environments.

### Addressing the transparency gap

Finally, beyond the challenges posed by infrastructural and data issues, the problem of transparency must be addressed. True scientific progress requires more than a succinct methods paragraph in an academic paper—it also requires accessible code, model weights, and even data-processing pipelines. There are understandable limitations on sharing medical data due to patient privacy concerns. However, as recent meta-research studies in the field of radiology attest, code- and model-sharing practices are also suboptimal - and even when code was shared, documentation was often poor^26,27^. This transparency gap limits reproducibility, meaningful replication and benchmarking, and bias testing. In addition, it prevents others from building on the original AI model or learning from its methods, which limits adaptation to new settings or reuse for related research questions.

Promoting open-source development is an essential first step toward addressing this gap. Initiatives such as RETFound^28^ and RETFound Green^29^, which are open-source ophthalmic foundation models, have demonstrated the value of releasing model architectures and pretrained weights for community use, enabling downstream fine-tuning and transfer learning across disease domains and use cases. Encouraging contributions from the wider research community beyond the original developers can transform code repositories into shared scientific resources that evolve through collective expertise. Modern frameworks such as vLLM (an open-source high-efficiency inference system for large language models) illustrate how transparent and collaborative community development can accelerate progress^30^.

However, this must also be balanced against the potential risks of model inversion (attempts to reconstruct sensitive attributes of the training data) and membership inference (attempts to determine whether an individual contributed training data) attacks, which are particularly pertinent to medical AI models^31,32^. Mitigation strategies such as privacy-preserving training methods (e.g. differential privacy)^33^ or data augmentation or regularisation to reduce memorisation can reduce these risks^32^, but typically involve trade-offs with performance, transparency, and reproducibility^33^. In addition, decisions about openness are also shaped by the fact that models developed by or in partnership with commercial entities constitute protected intellectual property, limiting direct adoption of idealised practices^34^. All these underscore the need for layered and domain-specific approaches to openness in medical AI, for example, through transparency of methods and assumptions when model weights cannot be shared.

## Retraining as scholarship

The case for prioritising model retraining and lifecycle management is therefore clear. While innovation in model architectures remains essential for advancing methodological frontiers and addressing tasks beyond the scope of current designs, doing so for tasks that could be addressed through retraining existing systems is not always intellectually, computationally, or environmentally efficient. Instead, with iterative updating, AI models can evolve from one-off artefacts into academic assets for different use cases. Each iteration can add to the collective knowledge of a model’s behaviour across diverse contexts. As such, rather than framing model retraining as mere maintenance, it could be considered as a form of scientific enquiry.

The focus expands from simply asking ‘Can an AI model perform a task?’ and ‘How well can it perform this task?’, to answering key questions about sample-efficiency for retraining, how retraining affects equity or bias, predicting model degradation, determining the threshold for retraining^35^, and techniques to be able to do this efficiently. Reframing model retraining as scholarship also means examining the methodological choices and validation practices that accompany model updates. For example, continual learning raises empirical questions about performance stability, the risk of catastrophic forgetting, and the governance of models that evolve outside fixed evaluation cycles^19,20^. In contrast, active learning raises design questions around when and how a model should seek human input, how these selections affect future data distributions, and how annotation strategies shape downstream performance. Adopting lifecycle practices such as transparent versioning of models, routine regression testing, and documented monitoring helps transform this process into shared, reproducible science and reduces technical debt to help accelerate progress robustly^36,37^. These two directions (retraining existing models and developing novel architectures) should be viewed as complementary rather than competing. A balanced research ecosystem should reward both the creation of new frameworks and the thoughtful stewardship of existing ones through transparent, well-documented lifecycle management.

Beyond the technical, data, and infrastructural barriers, the incentive structures of academic research may pose a further challenge to model retraining in healthcare. Researchers may think that grant proposals are more readily funded and high-impact publications more easily secured by developing novel and high-impact models, rather than undertaking the complex work of adapting and validating existing ones across different clinical contexts. This perception systemically disincentivises the long-term maintenance of AI models. It is the authors’ opinion that a conceptual shift towards considering model adaptability and retraining as a key part of scientific enquiry is crucial - this may help avoid the current proliferation of promising but ultimately brittle models, as well as the accumulation of technical debt across the field. Recent research attests to the value of this, with one study suggesting that continual learning approaches outperform episodic fine-tuning, and that both exceed the performance of the original locked model in terms of generalisability^38^.

## Case studies in model retraining

Our own ongoing work in leveraging AI tools for scientific discovery to treat blinding eye disease provides two salient case studies that illustrate some of these considerations.

**Fig. 3:**
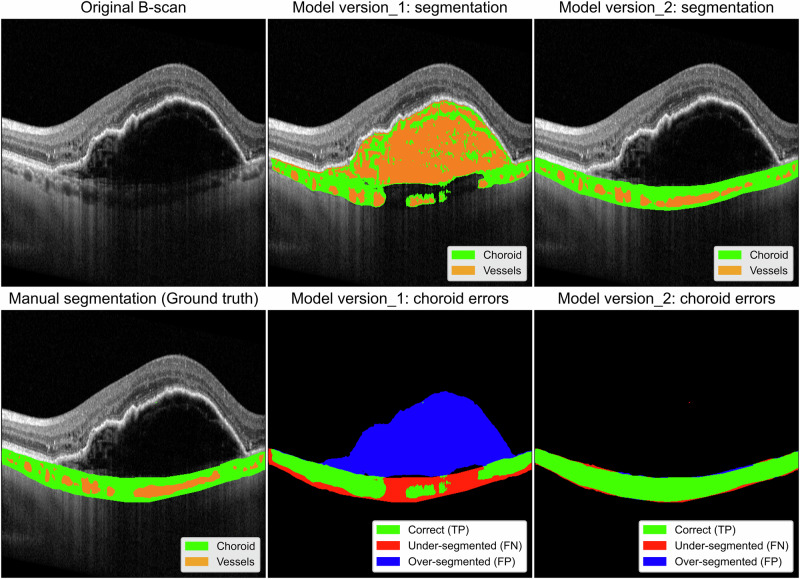
Example outputs from a deep learning model developed for segmenting choroidal vessels. This model was trained on healthy eyes, and did not perform well in eyes where pathology was present, such as with a large pigment epithelial detachment (PED) related to age-related macular degeneration. The model was fine-tuned on the newly annotated pathological scans. The panels in the first row show (from left to right) the original scan, segmentation from the original model (model version 1) and retrained model versions (model version 2), compared with the manually-labelled ground truth. The panels in the second row (from left to right) show the manual segmentation ground truth, followed by the comparisons between each version of the model with the ground truth. The original model features significant segmentation errors, having misinterpreted the PED as the choroid, while the retrained version (model version 2) demonstrates minimal variation from the ground truth. TP true positive, FP false positive, FN false negative.

Firstly, we sought to use an end-to-end pipeline developed by a collaborator for segmenting the choroid and choroidal vessels on macular optical coherence tomography (OCT) scans^39^. The model was trained and validated on a dataset of normal eyes, and performed its intended task with high fidelity in this population to produce quantitative metrics such as choroidal thickness, area, and vascular index. Our specific use case required the application of this model to eyes with different stages of age-related macular degeneration (AMD) (early, intermediate, advanced dry, advanced wet), whereupon significant performance degradation was seen, with some variability across each stage of disease (Fig. 3). This presented us not with a simple failure, but with a series of fundamental scientific questions that encapsulate some of the broader questions posed earlier: what is the minimum number of annotated AMD scans for each stage that is required to achieve robust performance? How do we retrain the model to recognise diseased anatomy without catastrophically forgetting its ability to segment healthy eyes? This offers an opportunity for scientific investigation into model resilience and capacity for generalisation—the very scholarship we argue is essential for bridging the AI chasm.

**Fig. 4:**
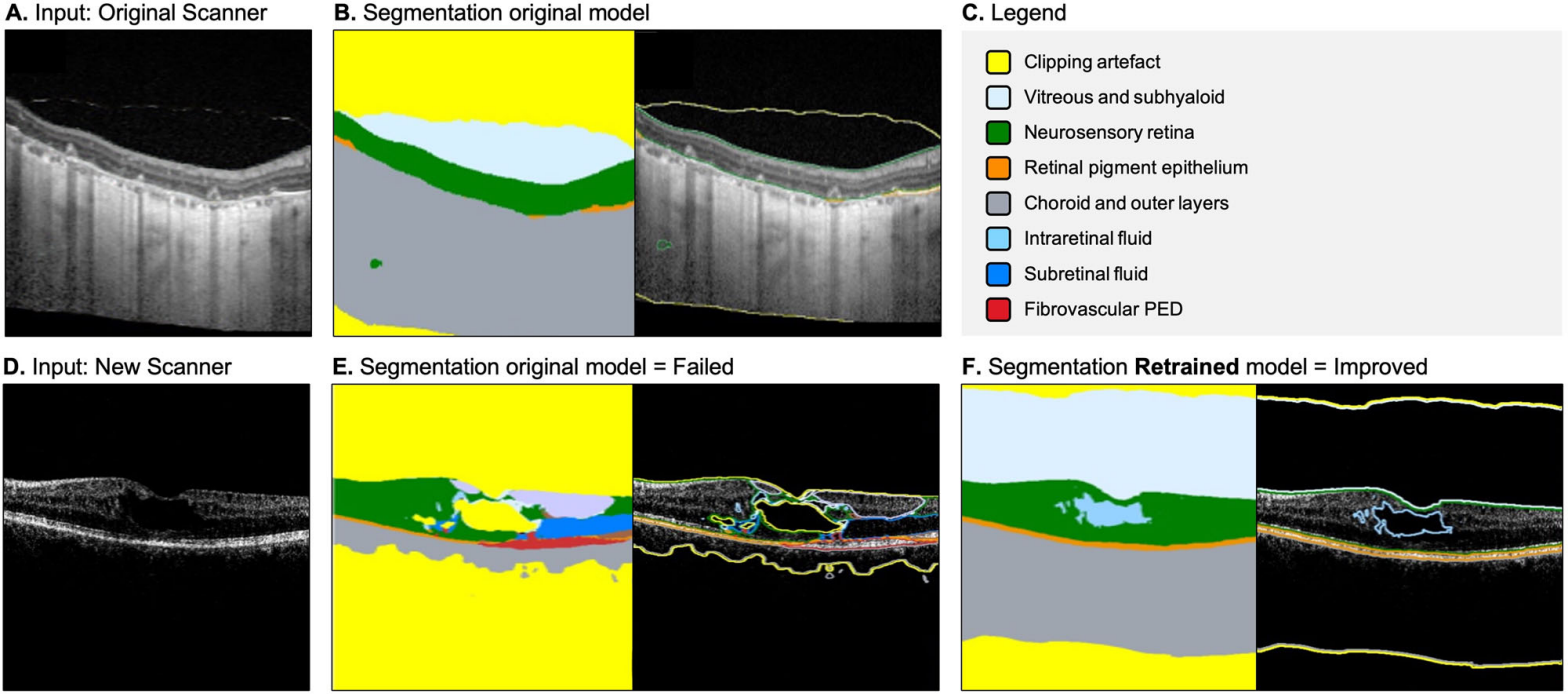
Example outputs from a deep learning model developed for segmenting retinal layers and pathological features on optical coherence tomography (OCT) scans of the macula. **A** An OCT scan coupled with accurate model segmentation outputs in (**B**)—note that the model was trained on scans from the same imaging device. **C** The annotation legend. **D** An OCT scan from an older imaging device with inaccurate model segmentation outputs in (**E**), followed by accurate segmentation outputs after the model was retrained (**F**). Retraining was performed using a ‘Noisy Student’ approach^42^ wherein the ‘student’ model learns from pseudo-labels generated by the original (‘Teacher’) model, with strong data augmentation introducing controlled noise that encourages robustness to distribution shift between imaging devices.

A further example is our adaptation of a deep learning model for segmenting retinal layers and pathological features on macular OCT scans, which was first trained close to a decade ago^40^. This model was trained on scans from a specific device manufacturer and model, and its performance deteriorated when tested on other device types (Fig. 4). This raised interesting questions about the nature of the domain shift and potential data harmonisation techniques to normalise these device-specific signatures without obscuring pathological features, as well as optimising the choice of model architecture and computational environment for retraining. The question of data efficiency arises as before: what is the minimal dataset required from a new device for effective fine-tuning, and can unsupervised domain adaptation techniques, such as a student-teacher approach (where the original model generates pseudo-labels for scans from the new device), bypass the need for re-annotation altogether?

Together, these examples illustrate that the choice of strategy is guided less by the source of dataset shift than by the extent and nature of performance degradation. In some cases, limited degradation can be addressed through targeted parameter updates. In others, more substantial mismatches between training and deployment data necessitate broader re-optimisation, including retraining via self-training approaches. Accordingly, both acquisition-level changes and population-level differences may necessitate anything from modest parameter adjustment to more extensive retraining, depending on the severity and manifestation of performance decline.

These examples also underscore how model retraining is not merely maintenance, but an essential component for achieving robust and generalisable medical AI. In either case, training a new model from scratch was regarded by our group as an inefficient non-starter given the prohibitive cost of expert annotation, data acquisition, and compute requirements. Having access to the base model and/or willing colleagues or collaborators was key to facilitating this process - one which is not always possible due to the logistical challenges of cross-institutional collaboration and the ‘publish or perish’ culture that can discourage open collaboration, leaving aside the commercial sensitivities from proprietary models altogether. Once completed, these exemplar models will be published as open source in order to advance open science.

## Towards an open and sustainable academic ecosystem

The twin challenges of generalisability and drift lead to an unavoidable conclusion - all models require some form of retraining if they are to remain useful and safe as functional tools in the long run. Ultimately, the maturation of medical AI as a discipline is predicated on the long-term viability and adaptability of these tools, which will be determined by our capacity to manage them across their entire lifecycle. It is essential for academia to evolve beyond a focus on developing innovative proofs-of-concept and static high-performance models alone to recognise model retraining as scholarship, and for journals and funders to incentivise and support the science of how this is undertaken. Adopting standard imaging formats, promoting the development of diverse public datasets, and sharing best practices and code would help support researchers or institutions, particularly where resources and capacity may be more limited. We call on stakeholders to consider not just how we build AI models, but also how we should retrain, maintain, and share them.

## Data Availability

Not applicable.

## References

[1] Secinaro, S., Calandra, D., Secinaro, A., Muthurangu, V. & Biancone, P. The role of artificial intelligence in healthcare: a structured literature review. *BMC Med. Inform. Decis. Mak.* **21**, 125 (2021). 33836752 10.1186/s12911-021-01488-9 PMC8035061

[2] Wang, H. et al. Scientific discovery in the age of artificial intelligence. *Nature* **620**, 47–60 (2023). 37532811 10.1038/s41586-023-06221-2

[3] Keane, P. A. & Topol, E. J. With an eye to AI and autonomous diagnosis. *npj Digit. Med.* **1**, 40 (2018). 31304321 10.1038/s41746-018-0048-y PMC6550235

[4] Youssef, A. et al. External validation of AI models in health should be replaced with recurring local validation. *Nat. Med.* 1–2 10.1038/s41591-023-02540-z (2023). 10.1038/s41591-023-02540-z 37853136

[5] Oduro-Gyan, J., Eleweke, I., Ajuwon, S., Bello, A. & Arotayo, A.-L. Embedding responsible AI into MLOps pipelines: ensuring fairness, explainability, and governance in KYC and FinTech decisioning. *J. Sci. Res. Rep.* **31**, 563–581 (2025).

[6] Reddi, V. J. Chapter 13: ML Operations. In *Machine Learning Systems: Principles and Practices of Engineering Artificially Intelligent Systems* https://mlsysbook.ai/book/.

[7] Rajagopal, A. et al. Machine learning operations in health care: a scoping review. *Mayo Clin. Proc. Digit. Health* **2**, 421–437 (2024). 40206123 10.1016/j.mcpdig.2024.06.009 PMC11975983

[8] Wilkinson, M. D. et al. The FAIR Guiding Principles for scientific data management and stewardship. *Sci. Data* **3**, 160018 (2016). 26978244 10.1038/sdata.2016.18 PMC4792175

[9] Mackenzie, A., Loveland, J. & van Engen, R. Survey of image processing settings used for mammography systems in the United Kingdom: How variable is it? Preprint at *arXiv.org* https://arxiv.org/abs/2501.13595v1; 10.1117/12.3026876 (2025).

[10] Lee, A. Y. et al. Recommendations for standardization of images in ophthalmology. *Ophthalmology* **128**, 969–970 (2021). 33832778 10.1016/j.ophtha.2021.03.003 PMC8335850

[11] Besson, S. et al. Bringing open data to whole slide imaging. *Digit. Pathol.* **2019**, 3–10 (2019). 10.1007/978-3-030-23937-4_1 PMC6774793 31579322

[12] Arnaout, R. et al. The (heart and) soul of a human creation: designing echocardiography for the big data age. *J. Am. Soc. Echocardiogr.* **36**, 800–801 (2023). 37191597 10.1016/j.echo.2023.04.016 PMC10913146

[13] Heidelberg Eye Explorer 2 (HEYEX 2)—Platform for Ophthalmic Image Management and Device Integration. Heidelberg Engineering. https://business-lounge.heidelbergengineering.com/us/en/products/heidelberg-eye-explorer/heyex-2/#product-details.

[14] Finlayson, S. G. et al. The clinician and dataset shift in artificial intelligence. *N. Engl. J. Med.* **385**, 283–286 (2021). 34260843 10.1056/NEJMc2104626 PMC8665481

[15] Subbaswamy, A., Chen, B. & Saria, S. A unifying causal framework for analyzing dataset shift-stable learning algorithms. *J. Causal Inference* **10**, 64–89 (2022).

[16] Lee, S., Yin, C. & Zhang, P. Stable clinical risk prediction against distribution shift in electronic health records. *Patterns* **4**, 100828 (2023). 10.1016/j.patter.2023.100828 PMC10499849 37720334

[17] Avati, A., Xue, E., Xu, Z., Lakshminarayanan, B. & Dai, A. BEDS-Bench: behavior of EHR-models under distributional shift—a benchmark. In *Proc. Workshop on Distribution Shifts, 35th Conference on Neural Information Processing Systems*10.48550/arXiv.2107.08189 (2021).

[18] De-Kayne, R. et al. Sequencing platform shifts provide opportunities but pose challenges for combining genomic data sets. *Mol. Ecol. Resour.* **21**, 653–660 (2021). 33314612 10.1111/1755-0998.13309

[19] Lee, C. S. & Lee, A. Y. Applications of continual learning machine learning in clinical practice. *Lancet Digit. Health* **2**, e279–e281 (2020). 33328120 10.1016/S2589-7500(20)30102-3 PMC8259323

[20] Chen, Z. & Liu, B. Lifelong Machine Learning. 10.1007/978-3-031-01581-6 (2018).

[21] Khan, S. M. et al. A global review of publicly available datasets for ophthalmological imaging: barriers to access, usability, and generalisability. *Lancet Digit. Health* **3**, e51–e66 (2021). 33735069 10.1016/S2589-7500(20)30240-5 PMC7618278

[22] Ong, A. Y. et al. A scoping review of artificial intelligence as a medical device for ophthalmic image analysis in Europe, Australia and America. *npj Digit. Med.* **8**, 1–13 (2025). 40442400 10.1038/s41746-025-01726-8 PMC12122805

[23] Ransohoff, D. F. & Feinstein, A. R. Problems of spectrum and bias in evaluating the efficacy of diagnosTIC TESTs. *N. Engl. J. Med.* **299**, 926–930 (1978). 692598 10.1056/NEJM197810262991705

[24] Ong, A. Y., Hogg, H. D. J. & Keane, P. A. Cochrane corner: artificial intelligence for diagnosing exudative age-related macular degeneration. *Eye* 1–2 10.1038/s41433-025-03599-3 (2025). 10.1038/s41433-025-03599-3 PMC11885809 39833576

[25] Engelmann, J., Storkey, A. & Bernabeu LLinares, M. Exclusion of poor quality fundus images biases health research linking retinal traits and systemic health. *Investig. Ophthalmol. Vis. Sci.* **64**, 2922 (2023).

[26] Venkatesh, K., Santomartino, S. M., Sulam, J. & Yi, P. H. Code and data sharing practices in the radiology artificial intelligence literature: a meta-research study. *Radio. Artif. Intell.* **4**, e220081 (2022). 10.1148/ryai.220081 PMC9530751 36204536

[27] Lee, T., Lee, J. H., Yoon, S. H., Park, S. H. & Kim, H. Availability and transparency of artificial intelligence models in radiology: a meta-research study. *Eur. Radiol.*10.1007/s00330-025-11492-6 (2025). 10.1007/s00330-025-11492-6 PMC12350510 40095011

[28] Zhou, Y. et al. A foundation model for generalizable disease detection from retinal images. *Nature* 1–8 10.1038/s41586-023-06555-x (2023). 10.1038/s41586-023-06555-x PMC10550819 37704728

[29] Engelmann, J. & Bernabeu, M. O. Training a high-performance retinal foundation model with half-the-data and 400 times less compute. *Nat. Commun.* **16**, 6862 (2025). 40715051 10.1038/s41467-025-62123-z PMC12297332

[30] vLLM. https://docs.vllm.ai/en/latest/.

[31] Hintersdorf, D., Struppek, L. & Kersting, K. Balancing Transparency and Risk: An Overview of the Security and Privacy Risks of Open-Source Machine Learning Models. In Bridging the Gap Between AI and Reality. AISoLA 2023. Lecture Notes in Computer Science, (eds Steff en, B.) Vol. 14129 (Springer, Cham, 2023).

[32] Wei, J. et al. Memorization in deep learning: a survey. *ACM Comput. Surv.* **58**, 98:1–98:35 (2025).

[33] Mohammadi, M. et al. Differential privacy for medical deep learning: methods, tradeoffs, and deployment implications. *npj Digit. Med.* **9**, 93 (2026). 10.1038/s41746-025-02280-z PMC12855931 41484344

[34] Heil, B. J. et al. Reproducibility standards for machine learning in the life sciences. *Nat. Methods* **18**, 1132–1135 (2021). 34462593 10.1038/s41592-021-01256-7 PMC9131851

[35] Mahadevan, A. & Mathioudakis, M. Cost-aware retraining for machine learning. *Knowl.-Based Syst.* **293**, 111610 (2024).

[36] Sculley, D. et al. Hidden technical debt in machine learning systems. In *Proc. Advances in Neural Information Processing Systems* Vol. 28 (Curran Associates, Inc., 2015).

[37] Serban, A., van der Blom, K., Hoos, H. & Visser, J. Software engineering practices for machine learning—adoption, effects, and team assessment. *J. Syst. Softw.* **209**, 111907 (2024).

[38] Chen, E. et al. International retrospective observational study of continual learning for AI on endotracheal tube placement from chest radiographs. *NEJM AI* **3**, AIoa2500522 (2025).

[39] Engelmann, J. et al. Choroidalyzer: an open-source, end-to-end pipeline for choroidal analysis in optical coherence tomography. *Investig. Ophthalmol. Vis. Sci.* **65**, 6 (2024). 10.1167/iovs.65.6.6 PMC11156207 38833259

[40] De Fauw, J. et al. Clinically applicable deep learning for diagnosis and referral in retinal disease. *Nat. Med.* **24**, 1342–1350 (2018). 30104768 10.1038/s41591-018-0107-6

[41] Tazbaz, T. & Nicol, J. Blog: a lifecycle management approach toward delivering safe, effective AI-enabled health care. FDA. https://www.fda.gov/medical-devices/digital-health-center-excellence/blog-lifecycle-management-approach-toward-delivering-safe-effective-ai-enabled-health-care.

[42] Xie, Q., Luong, M.-T., Hovy, E. & Le, Q. V. Self-training with Noisy Student improves ImageNet classification. *2020 IEEE/CVF Conference on Computer Vision and Pattern Recognition (CVPR)*, 10684–10695, (Seattle, WA, USA, 2020).

